# A Proactive Environmental Approach for Preventing Legionellosis in Infants: Water Sampling and Antibiotic Resistance Monitoring, a 3-Years Survey Program

**DOI:** 10.3390/healthcare7010039

**Published:** 2019-03-08

**Authors:** Ioanna Alexandropoulou, Theodoros Parasidis, Theocharis Konstantinidis, Maria Panopoulou, Theodoros C. Constantinidis

**Affiliations:** 1 Laboratory of Hygiene and Environmental Protection, Medical School, Democritus University of Thrace, Campus (Dragana) Building 5, 68131 Alexandroupolis, Greece; tparasid@med.duth.gr (T.P.); tkonsta@med.duth.gr (T.K.); tconstan@med.duth.gr (T.C.C.); 2 Microbiology Laboratory, Medical School, Democritus University of Thrace, Campus (Dragana), 68131 Alexandroupolis, Greece; mpanopou@med.duth.gr

**Keywords:** infant, *Legionella* spp., environmental monitoring, waterborne pathogens, antibiotic, e-test, water distribution system, health care facilities, public health

## Abstract

A proactive environmental monitoring program was conducted to determine the risk and prevent nosocomial waterborne infections of *Legionella* spp. in infants. Sink taps in a neonatal intensive care unit (NICU) and two obstetric clinics were monitored for *Legionella* spp. A total of 59 water samples were collected during a 3-year period and 20 of them were found colonized with *Legionella pneumophila*. Standard culture, molecular, and latex agglutination methods were used for the detection and identification of *Legionella* bacteria. Hospital personnel also proceeded with remedial actions (hyperchlorination and thermal shock treatment) in the event of colonization. The minimal inhibitory concentration (MIC) values of erythromycin, ciprofloxacin was determined for *Legionella* isolates using the e-test method. Our data indicate that the majority of neonatal sink-taps were colonized at least once during the study with *Legionella* spp. Among 20 isolates, 5 were considered as low-level resistant, 3 in erythromycin and 2 in ciprofloxacin, while no resistant strains were detected. Environmental surveillance in neonatal and obstetric units is suggested to prevent waterborne infections, and thus to reduce the risk of neonatal nosocomial infections.

## 1. Introduction

*Legionella* bacteria are ubiquitous in aquatic natural or artificial environments and in soil, while the main route of transmission is through aspiration of colonized water [1]. In health care facilities, water distribution systems, including tap water, cooling towers, humidifiers, and fountains, are the main source of infection [2]. These bacteria are the etiologic agent for Legionnaires’ disease (LD) and Pontiac fever; both also called legionellosis, and are the second most common cause for intensive cares unit hospitalization [3,4]. 

According to the European Center for Disease Prevention and Control (ECDC), the rate of Legionnaires’ disease in Europe for 2017 was 18 cases per million inhabitants. Greece reported 43 legionellosis cases, all confirmed. During the same period, 9 out of the 30 countries reporting to ECDC reported 28 legionellosis outbreaks, either community or hospital acquired [5]. During 2011–2015, 4.9% of all legionellosis cases reported in the European Union or European Economic Area were possibly hospital acquired, while another 2.4% had different health-care facilities as a probable source of infection [6].

LD is rare in children and infants, although cases are reported in literature, mainly due to colonized water distribution systems, or other water devices in hospitals [7,8]. Another major source of infection, especially for neonates, is water birth pools [9].

Pharmaceutical treatment is based on the use of antibiotics, macrolides, and flouroquinolones [10]. Erythromycin is the first line drug in the treatment of legionellosis, even though resistant strains can easily be obtained in vitro [11,12]. Flouroquinolones has been shown to survive high intracellular inhibition of *Legionella* compared with erythromycin [13,14]. Despite this, it was found that strain resistance to fluoroquinolones can also be easily achieved in vitro [15]. 

In this study we describe a proactive environmental program followed by two obstetrics clinics (Obstetric clinic II and Obstetric clinic III) and one neonatal intensive care unit (NICU), along with the remedial actions that they were taken when colonization occurred. The goal was to prevent infant legionellosis by decreasing or even eliminate *Legionella* spp. colonization. All three clinics are located in three different health care facilities in our region. In addition, in order to estimate the antibiotic susceptibility of the isolated *Legionella* bacteria, we proceeded with in vitro antibiotic resistance monitoring, determining minimal inhibitory concentration (MIC) values of erythromycin and ciprofloxacin against all isolates using e-test on Buffered Charcoal Yeast Extract (BCYE) with L-cysteine agar.

## 2. Materials and Methods 

Water Samplings: The water samplings were performed by personnel of the Laboratory of Hygiene and Environmental Protection trained hospital staff according to ISO 19458:2006, under the recommendations and authorized permission of each health care facility’s authorities [16]. Samples were taken with previous flushing and without previous tap disinfection. At each sampling location, a volume of 0.5 L was collected in a glass container, which had been sterilized in an autoclave (120 °C 15 min., 1 atm pressure) to ensure the absence of viable microorganism. The samples were stored at 4 °C and processed within 24 h. In NICU, eight environmental samplings were conducted between July 2007 and December 2010. In Obstetrics Clinics II and III, six samplings took place between March 2008 and July 2010, and between April 2008 and June 2010, respectively. 

Sample analysis and culture: A volume of 0.5 L of each sample was analyzed for *Legionella* spp., according to standard method W12 [17]. The procedures are based on ISO 11731 [18] and is especially used in water samples from hot and cold water systems in large buildings. Briefly, after filtration and centrifugation, 1 ml of the supernatant was kept to re-suspend the deposit. Decimal dilutions from the untreated stored concentrate were also used. Each sample was triple plated onto Legionella GVPC agar (Oxoid): (a) 0.1 mL of concentrate without any treatment, (b) 0.1 mL of concentrate after heat treatment at 50 °C for 30 min, and (c) 0.2 mL of the concentrate after acid treatment with a double strength acid buffer pH 2.2. The plates were sealed in bags and incubated at 37 °C for 10 days. Typical legionella–like bacteria colonies were subcultured to BCYE with L-cysteine agar (Oxoid) and BCYE without L-cysteine agar (Oxoid) and incubated for at least 2 days. Colonies were identified as *Legionella pneumophila* (serogroup 1 and 2–15) or *Legionella* spp. using latex agglutination tests (Legionella Slidex test kit, Biomerieux, and Legionella latex kit, Oxoid). Each isolate was subcultured once more before storage at −80 °C. The detection limit in our method was 10 cfu/lt.

DNA extraction: Bacterial DNA from colonies was extracted with the Dneasy Blood and Tissue kit (Qiagen), according to the manufacturer’s instructions.

PCR analysis: The primers LmipL920 (5’GCTACAGACAAGGATAAGTTG 3’) and LmipR1548 (5’GTTTTGTATGACTTTAATTCA 3’) targeting a 650-bp region in the *mip* gene were used for the specific detection of *Legionella pneumophila* (650-bp product), as was described before [19]. Briefly, a 50 μL reaction mixture was used, containing 3U of Taq DNA polymerase (BioTaq, Bioline) with the supplied PCR buffer, 0.2 mM of primers LmipL920 and LmipR1548, 0.2 mM of each deoxynucleoside triphosphate, and 3 mM of MgCl_2_. Amplification was performed in a Mastercycler gradient (Eppendorf) with a PCR thermal profile consisting of an initial incubation for 2 min at 94 °C, 40 cycles of 20 s at 94 °C, 30 s at 60 °C, and 40 s at 72 °C, and finally a postamplification step of 2 min at 72 °C. Subsequently, PCR products were analyzed by 2% agarose gel electrophoresis.

E-test analysis: E-test analysis proceeded as in previous studies [20,21]. Because there are no official breakpoints for *Legionella* spp., in this study we used the National Committee for Clinical Laboratory Standards (NCCLS) guidelines [18]. Based on these guidelines, bacteria are considered susceptible (S) to ciprofloxacin when the MIC values are ≤ 1 μg/mL and resistant (R) when there are ≥ 4 μg/mL, and for erythromycin these values are S ≤ 0.5 μg/mL and R ≥ 8 μg/mL, respectively [21].

Bacterial reference strain: The following reference strain *Legionella pneumophila* serogroup 1 National Collection of Type Cultures (NCTC) 12821 (Health Protection Agency, HPA) was used as a control during DNA extraction, PCR amplification, and e-test. DNA-free water was analyzed in each experiment to check for possible DNA contamination during filtration, DNA extraction, and PCR amplification.

## 3. Results

A total of 59 water samples were collected. In the event of colonization, health care’s facilities training staff proceeded to remedial actions—thermal shock disinfection and chemical disinfection when hot water systems and cold water systems were colonized with legionella bacteria, respectively (Table 1, Table 2 and Table 3).

A total of 20 samples were colonized with *Legionella* spp. We isolated 20 Legionella bacteria strains (each positive sample revealed only one isolate), which all belonged to Legionella pneumophila species according to PCR. Latex agglutination test showed that isolates belonged to Legionella pneumophila serogroup 1 and Legionella pneumophila serogroup 2–15 for NICU and Obstetric clinics II and III, respectively (Tables S1, S2, S3).

The susceptibility range was 0.04–2 mg/L for erythromycin and 0.04–1 mg/L for ciprofloxacin. Three and two isolates were considered as low-level resistant in erythromycin and in ciprofloxacin, respectively (Table 4). No resistant strains were detected.

## 4. Discussion

In order to assess the risk of neonatal legionellosis in our region, we cooperated with our local health care facilities’ authorities and conducted a proactive environmental surveillance program for Legionella detection specifically focused on neonatal and obstetrics units. We also screened the isolated strains for antibiotic susceptibility in vitro, determining the minimal inhibitory concentration (MIC) values of erythromycin and ciprofloxacin using the e-test system.

During our study there was no specific legislation for the detection of *Legionella* spp. in Greece, except of a series of guidelines, demonstrated by the Hellenic Ministry of Health. These were conducted on the basis of European Working Group for Legionella Infections (EWGLI) instructions [22]. Since 2017, the new legislation on monitoring the quality of human water included in Government Gazette 3282/Β/19-9-2017, which is based on Directive (EC) No 2015/1787/EU, determines the monitoring of legionella in water distribution systems of health-care facilities and nursing homes, tourist facilities, hotels, prisons, and camps with a parametric value of 1000 cfu/1L [23]. The minimum frequency is twice a year and the responsibility for the analysis lies on the authorities of the buildings. 

Even though infant legionellosis is rare, cases are reported in literature and almost all of them are linked to a colonized water source. In a previous paper we reviewed cases of neonatal and child legionellosis [24]. 

Collins et al. report a case of LD after water birth at home with a pool filled before labor. *Legionella pneumophila* ST48 was detected both in the neonate’s samples and in the pool [25]. Another case, probable linked to the pool water, was an 8-day old infant with multi-organ failure and *Legionella pneumophila* serogroup 6 infection [26]. A fatal case was reported in 2014 concerning a 25-day old neonate with *Legionella pneumophila* serogroup 1 infection after water birth [27]. Another two cases took place in 2016 in Arizona after water birth at home. Both were infected with *Legionella pneumophila*, serogroup 1, and serogroup 6, respectively. The first one also had a congenital heart disease [28]. There was a *Legionella pneumophila* serotype 1 and aspergillus infection in a child almost 2 years old with T-lymphoblastic leukemia who was probably exposed to apparatus discharging water after showering [29].

Our study demonstrated that the majority of the neonatal sink taps water was colonized with *Legionella* spp. at least once, although different levels of colonization was revealed in each one (Tables S1, S2, S3 supplementary material). Especially in Obstetrics clinics II and III, sink taps had higher concentrations of *Legionella* bacteria during the study time period. Remedial actions (thermal shock treatment and hyperchlorination) taken by the health care facility staff reduced or eliminated colonization, although in most cases only temporarily, as water was found recolonized during the next sampling round (Table 1, Table 2 and Table 3). An explanation is that *Legionella* spp. is ubiquitous in aquatic systems, thus, *Legionella* strains can very often enter the water system, and under factors that favor their multiplication, such as biofilm formation, stagnation, and water temperature, can multiply and reach high levels [30]. Thus, constant environmental surveillance is crucial in order to prevent high levels of colonization. 

Controlling *Legionella* bacteria in hospital water systems is still the main preventive measure. Thus, new strategies for reducing or eliminating contamination in taps and pipelines are crucial. Casini et al. describe a new treatment with hydrogen peroxide and food-grade polyphosphates at 25 mg/L, which can even eliminate colonization after retaining residuals levels stable [31]. Another promising method is the installation of time flow taps (TFTs) in hospital settings near dead end branches to avoid biofilm formation [32].

In NICU, the health care facility’s authorities decided to install point of-use filters at the sink taps. This remedial action is not uncommon, but it has the limitation that filters should be replaced regularly [33]. During the surveillance program we also analyzed water samples from the filters a week after their replacement and found no positive samples.

As there are no specific recommendations, in the present study we use the e-test method, because it is simpler, less laborious, and it can be used as a routine procedure in most laboratories [34].

In the present study we choose to evaluate the in vitro MIC of strains to erythromycin and a very common fluoroquinolone antibiotic, ciprofloxacin. All the isolates were inhibited by low concentrations of the two antibiotics tested, except strains S35, S55, S57, S13, and S23, which were considered as low-level resistant in erythromycin and in ciprofloxacin, respectively. Acquisition of antibiotic resistance from the environment is not uncommon for *Legionella* bacteria, due to their exposure to medically or veterinary wastewater [35]. Low antibiotic susceptibility of environmental strains could raise the risk of patients’ treatment failure after infection, thus increasing morbidity and mortality rates. Constant raising of awareness from doctors and other health-care professionals is imperative to prevent infections acquired in hospital environments.

## 5. Conclusions

Our results highlight the risk for *Legionella* bacteria transmission to infants and also highlights the need for intensive environmental monitoring of waterborne pathogens in hospital settings. Continuous surveillance for infections acquired in hospital environments is the most appropriate prevention method for pathogens, such as *Legionella* bacteria. 

## Figures and Tables

**Table 1:** Remedial actions taken in the event of contamination in NCIU, water samples in each sampling round, and *Legionella pneumophila* cfu range.

Remedial Action after Sampling	Sampling Round	Year	Number of Contaminated Samples/Number of Samples	*L. pneumophila* cfu Range
	1st	2007	0/4	-
No treatment				
	2nd	2007	3/3	1000–1600
Thermal shock treatment	All three colonized samples were hot water samples, so thermal shock treatment was used at 70–80 °C for a short period.			
	3rd	2008	0/3	-
No treatment				
	4th	2008	0/2	-
No treatment				
	5th	2009	2/4	100–120
Filter	Installation of filters in one sink tap			
	6th	2009	0/2	-
No treatment				
	7th	2010	2/5	2000–21,500
Thermal shock treatment	Both colonized samples were hot water samples, so thermal shock treatment was used at 70–80 °C for a short period.			
	8th	2010	1/6	1000
Thermal shock treatment	One colonized hot water sample was detected, so thermal shock treatment was used at 70–80 °C for a short period.			

**Table 2:** Water samples in each sampling round, *Legionella pneumophila* serogroup/cfu range, and remedial actions taken in the event of contamination in Obstetrics clinic II.

Remedial Action after Sampling	Sampling Round	Year	Number of Contaminated Samples/Number of Samples	*L. pneumophila* cfu Range
	1st	2008	2/2	1040–1200
Thermal shock treatment/hyperchlorination	Hot water sample: thermal shock treatment was used at 70–80 °C for a short period. Cold water sample: Shock hyperchlorination was conducted by adding high concentrations of chlorine into the system for a couple of hours			
	2nd	2008	0/4	-
No treatment				
	3rd	2009	0/4	-
No treatment				
	4th	2009	2/2	28,000–38,000
Thermal shock treatment/hyperchlorination	Hot water sample: thermal shock treatment was used at 70–80 °C for a short period. Cold water sample: Shock hyperchlorination was conducted by adding high concentrations of chlorine into the system for a couple of hours			
	5th	2010	2/2	16,500–23,500
Thermal shock treatment/hyperchlorination	Hot water sample: thermal shock treatment was used at 70–80 °C for a short period. Cold water sample: Shock hyperchlorination was conducted by adding high concentrations of chlorine into the system for a couple of hours			
	6th	2010	0/2	-
No treatment				

**Table 3:** Water samples in each sampling round, *Legionella pneumophila* serogroup/cfu range and remedial actions taken in the event of contamination in Obstetrics clinic III.

Remedial Action after Sampling	Sampling Round	Year	Number of Contaminated Samples/Number of Samples	*L. pneumophila* cfu Range
	1st	2008	1/2	8000
Thermal shock treatment	One colonized hot water sample was detected, so thermal shock treatment was used at 70–80 °C for a short period.			
	2nd	2008	1/2	2000
Thermal shock treatment	One colonized hot water sample was detected, so thermal shock treatment was used at 70–80 °C for a short period.			
	3rd	2009	2/3	320–5600
Thermal shock treatment	Two of the three hot water sample were colonized, so thermal shock treatment was used at 70–80 °C for a short period.			
	4th	2009	1/2	31,500
Thermal shock treatment	One colonized hot water sample was detected, so thermal shock treatment was used at 70–80 °C for a short period.			
	5th	2010	0/2	-
No treatment				
	6th	2010	1/2	51,800
Thermal shock treatment	One colonized hot water sample was detected, so thermal shock treatment was used at 70–80 °C for a short period.			

**Table 4:** Minimal Inhibitory Concentration (MIC) values of *Legionella pneumophila* isolates.

**Erythromycin MIC mg/L**	**0.016**	**0.023**	**0.23**	**0.25**	**0.38**	**0.5**	**0.75**	**1**
*L. pneumophila* isolates	S5,S6,S7,S23, S31,S37,S39,	S13, S14, S42, S44	S24, S56	S32	S22	S45, S54	S35	S55, S57
**Ciprofloxacin MIC mg/L**	**0.04**	**0.08**	**0.094**	**0.19**	**0.25**	**0.5**	**1**	**2**
*L. pneumophila* isolates	S5,S6,S7,S24	S42,S44, S56	S32	S31,S37, S39,S55	S45, S54	S14, S22, S57	S35	S13, S23

## Supplementary Materials

The following are available online at https://www.mdpi.com/2227-9032/7/1/39/s1, Table S1: Water samples in each sampling round, remedial actions taken in the event of contamination and *Legionella pneumophila* isolates in NCIU. Table S2: Water samples in each sampling round, remedial actions taken in the event of contamination and *Legionella pneumophila* isolates in Obstetrics clinic II. Table S3: Water samples in each sampling round, remedial actions taken in the event of contamination and *Legionella pneumophila* isolates in Obstetrics clinic III.
