# Supplementary material for: A Proactive Environmental Approach for Preventing Legionellosis in Infants: Water Sampling and Antibiotic Resistance Monitoring, a 3-Years Survey Program
Source: Healthcare (Basel). 2019 Mar 8;7(1):39. doi: 10.3390/healthcare7010039 (PMC6473578; doi:10.3390/healthcare7010039)
Supplement: Supplementary file 1 [file healthcare-07-00039-s001.pdf]

# Supplementary Material

# A Proactive Environmental Approach for Preventing Legionellosis in Infants: Water Sampling and Antibiotic Resistance Monitoring, a 3-Years Survey Program

**Ioanna Alexandropoulou, Theodoros Parasidis, Theocharis Konstantinidis, Maria Panopoulou and Theodoros C. Constantinidis**

***Table 1.*** Water samples in each sampling round, remedial actions taken in the event of contamination and Legionella pneumophila isolates in NCIU.

| Samples | Sampling round | Year | Clinic | Sample site | Cold / Hot water | *L. pneumophila* (cfu/L) | *L. pneumophila* serogroup |
|---|---|---|---|---|---|---|---|
| S1 | 1st | 2007 | NCIU | sink tap | hot | 0 | 0 |
| S2 | 1st | 2007 | NCIU | sink tap | hot | 0 | 0 |
| S3 | 1st | 2007 | NCIU | sink tap | hot | 0 | 0 |
| S4 | 1st | 2007 | NCIU | sink tap | hot | 0 | 0 |
| S5 | 2nd | 2007 | NCIU | sink tap | hot | 1.600 | 1 |
| S6 | 2nd | 2007 | NCIU | sink tap | hot | 1.000 | 1 |
| S7 | 2nd | 2007 | NCIU | sink tap | hot | 1.400 | 1 |
| **Remedial action: thermal shock treatment** | | | | | | | |
| S8 | 3rd | 2008 | NCIU | sink tap | hot | 0 | 0 |
| S9 | 3rd | 2008 | NCIU | sink tap | hot | 0 | 0 |
| S10 | 3rd | 2008 | NCIU | sink tap | hot | 0 | 0 |
| S11 | 4th | 2008 | NCIU | sink tap | cold | 0 | 0 |
| S12 | 4th | 2008 | NCIU | sink tap | hot | 0 | 0 |
| S13 | 5th | 2009 | NCIU | sink tap | cold | 120 | 1 |
| S14 | 5th | 2009 | NCIU | sink tap | hot | 100 | 1 |
| **Installation of filters in one sink tap** | | | | | | | |
| S15 | 5th | 2009 | NCIU | sink tap with filter | cold | 0 | 0 |

| | | | | | | | |
|---|---|---|---|---|---|---|---|
| S16 | 5th | 2009 | NCIU | sink tap with filter | hot | 0 | 0 |
| S17 | 6th | 2009 | NCIU | sink tap | hot | 0 | 0 |
| S18 | 6th | 2009 | NCIU | sink tap | cold | 0 | 0 |
| S19 | 7th | 2010 | NCIU | sink tap with filter | hot | 0 | 0 |
| S20 | 7th | 2010 | NCIU | sink tap | hot | 0 | 0 |
| S21 | 7th | 2010 | NCIU | sink tap with filter | hot | 0 | 0 |
| S22 | 7th | 2010 | NCIU | sink tap | hot | 2.000 | 1 |
| S23 | 7th | 2010 | NCIU | sink tap | hot | 21.500 | 1 |
| **Remedial action: thermal shock treatment** | | | | | | | |
| S24 | 8th | 2010 | NCIU | sink tap | hot | 1000 | 1 |
| S25 | 8th | 2010 | NCIU | sink tap | cold | 0 | 0 |
| S26 | 8th | 2010 | NCIU | sink tap with filter | hot | 0 | 0 |
| S27 | 8th | 2010 | NCIU | sink tap with filter | cold | 0 | 0 |
| S28 | 8th | 2010 | NCIU | sink tap with filter | hot | 0 | 0 |
| S29 | 8th | 2010 | NCIU | sink tap | hot | 0 | 0 |
| **Remedial action: thermal shock treatment** | | | | | | | |

***Table 2.*** Water samples in each sampling round, remedial actions taken in the event of contamination and Legionella pneumophila isolates in Obstetrics clinic II.

| Samples | Sampling round | Year | Clinic | Sample site | Cold / Hot water | *L. pneumophila* (cfu/L) | *L. pneumophila* serogroup |
|---|---|---|---|---|---|---|---|
| S44 | 1st | 2008 | Obstetrics clinic II | sink tap | cold | 1.040 | 2-15 |
| S45 | 1st | 2008 | Obstetrics clinic II | sink tap | hot | 1.200 | 2-15 |
| | | | **Remedial action: thermal shock treatment/ hyperchlorination** | | | | |
| S46 | 2nd | 2008 | Obstetrics clinic II | sink tap | cold | 0 | 0 |
| S47 | 2nd | 2008 | Obstetrics clinic II | sink tap | hot | 0 | 0 |
| S48 | 2nd | 2008 | Obstetrics clinic II | sink tap | cold | 0 | 0 |
| S49 | 2nd | 2008 | Obstetrics clinic II | sink tap | hot | 0 | 0 |
| S50 | 3rd | 2009 | Obstetrics clinic II | sink tap | cold | 0 | 0 |
| S51 | 3rd | 2009 | Obstetrics clinic II | sink tap | hot | 0 | 0 |
| S52 | 3rd | 2009 | Obstetrics clinic II | sink tap | cold | 0 | 0 |
| S53 | 3rd | 2009 | Obstetrics clinic II | sink tap | hot | 0 | 0 |
| S54 | 4th | 2009 | Obstetrics clinic II | sink tap | cold | 28.000 | 2-15 |
| S55 | 4th | 2009 | Obstetrics clinic II | sink tap | hot | 38.000 | 2-15 |
| | | | **Remedial action: thermal shock treatment/ hyperchlorination** | | | | |
| S56 | 5th | 2010 | Obstetrics clinic II | sink tap | hot | 23.500 | 2-15 |

***Table 3.*** Water samples in each sampling round, remedial actions taken in the event of contamination and Legionella pneumophila isolates in Obstetrics clinic III.

| Samples | Sampling round | Year | Clinic | Sample site | Cold / Hot water | *L. pneumophila* (cfu/L) | *L. pneumophila* serogroup |
|---|---|---|---|---|---|---|---|
| S30 | 1st | 2008 | Obstetrics clinic III | sink tap | cold | 0 | 0 |
| S31 | 1st | 2008 | Obstetrics clinic III | sink tap | hot | 8.000 | 2-15 |
| **Remedial action: thermal shock treatment** | | | | | | | |
| S32 | 2nd | 2008 | Obstetrics clinic III | sink tap | hot | 2.000 | 2-15 |
| S33 | 2nd | 2008 | Obstetrics clinic III | sink tap | hot | 0 | 0 |
| **Remedial action: thermal shock treatment** | | | | | | | |
| S35 | 3rd | 2009 | Obstetrics clinic III | sink tap | hot | 5.600 | 2-15 |
| S36 | 3rd | 2009 | Obstetrics clinic III | sink tap | cold | 0 | 0 |
| S37 | 3rd | 2009 | Obstetrics clinic III | sink tap | hot | 320 | 2-15 |
| **Remedial action: thermal shock treatment** | | | | | | | |
| S38 | 4th | 2009 | Obstetrics clinic III | sink tap | cold | 0 | 0 |
| S39 | 4th | 2009 | Obstetrics clinic III | sink tap | hot | 31.500 | 2-15 |
| **Remedial action: thermal shock treatment** | | | | | | | |
| S40 | 5th | 2010 | Obstetrics clinic III | sink tap | cold | 0 | 0 |
| S41 | 5th | 2010 | Obstetrics clinic III | sink tap | hot | 0 | 0 |
| S42 | 6th | 2010 | Obstetrics clinic III | sink tap | hot | 51.800 | 2-15 |
| S43 | 6th | 2010 | Obstetrics clinic III | sink tap | cold | 0 | 0 |
| **Remedial action: thermal shock treatment** | | | | | | | |
